# Promotion of mammalian angiogenesis by neolignans derived from soybean extracellular fluids

**DOI:** 10.1371/journal.pone.0196843

**Published:** 2018-05-08

**Authors:** Farzaneh Kordbacheh, Thomas J. Carruthers, Anna Bezos, Marie Oakes, Lauren Du Fall, Charles H. Hocart, Christopher R. Parish, Michael A. Djordjevic

**Affiliations:** 1 Department of Plant Sciences, Research School of Biology, Australian National University, Canberra, Australian Capital Territory, Australia; 2 ACRF Department of Cancer Biology and Therapeutics, The John Curtin School of Medical Research, Australian National University, Canberra, Australian Capital Territory, Australia; 3 Research School of Chemistry, Australian National University, Canberra, Australian Capital Territory, Australia; Medical College of Wisconsin, UNITED STATES

## Abstract

Excessive or insufficient angiogenesis is associated with major classes of chronic disease. Although less studied, small molecules which can promote angiogenesis are being sought as potential therapeutics for cardiovascular and peripheral arterial disease and stroke. Here we describe a bioassay-directed discovery approach utilising size exclusion and liquid chromatography to purify components of soybean xylem sap that have pro-angiogenic activity. Using high resolution accurate mass spectrometry and nuclear magnetic resonance spectroscopy, the structure of two pro-angiogenic molecules (***FK1*** and ***FK2***) were identified as *erythro*-guaiacylglycerol-8-O-4'-(coniferyl alcohol) ether (*e*GGCE), and *threo*-guaiacylglycerol-8-O-4'-(coniferyl alcohol) ether (*t*GGCE). These two molecules, which are coniferyl neolignan stereoisomers, promoted *in vitro* angiogenesis in the μM to nM range. Independently sourced samples of *e*GGCE and *t*GGCE exhibited comparable pro-angiogenic activity to the soybean derived molecules. The cellular mode of action of these molecules was investigated by studying their effect on endothelial cell proliferation, migration, tube formation and adhesion to the extracellular matrix (ECM) components, fibronectin and vitronectin. They were found to enhance endothelial cell proliferation and endothelial cell tube formation on Matrigel, but did not affect endothelial cell migration or adhesion to fibronectin and vitronectin. Thus, this study has identified two coniferyl neolignan stereoisomers, *e*GGCE and *t*GGCE, as pro-angiogenic molecules, with *e*GGCE being less active than *t*GGCE.

## Introduction

The formation of new capillaries from pre-existing vascular networks (angiogenesis) is a tightly controlled process in adult mammals. Excessive or insufficient angiogenesis is associated with major classes of chronic disease. Excessive angiogenesis is associated with cancer, psoriasis, age-related macular degeneration and arthritis [1–3] and this has fostered the isolation, identification and testing of an extensive range of anti-angiogenic molecules in clinical trials [4–6]. Although receiving less attention, the possibility of using pro-angiogenic molecules as therapeutic agents has recently emerged. Pro-angiogenic drugs have the potential to aid in treating diseases and conditions where the formation of new blood vessels is needed as, for example, in wound healing [1, 7, 8], cardiovascular disease [9, 10] and stroke [11–13]. Only a limited number of pro-angiogenic molecules have been studied in clinical settings, including erythropoietin (EPO) [14], β-carotene [15], nicotine [16–18], ferulic acid [19], stromal cell-derived factor 1 (SDF-1) [20] and VEGF [21]. However, unlike anti-angiogenic drug candidates, none of these molecules have translated well into the clinic [21, 22] and, therefore, it is of great interest to identify and explore more compounds with pro-angiogenic activity.

Historically, plant-derived natural products have been a rich source of therapeutic compounds, for example, salicylic acid [23], taxol [24], vincristine, vinblastine [5] and camptothecin [25] to name but a few. Recently Wang et al., scrutinised the available literature on plant-derived anti-angiogenic products and came up with a few phytochemical compounds which could affect tumour angiogenesis and pathological tumour vasculature [26]. However, pharmacologically active compounds in plants often exist at very low concentrations and this presents challenges to developing effective extraction and purification protocols [27].

A stratagem that enhances the potential for success in identifying potential therapeutic activities in fractionated plant material is to couple purification with a sensitive and robust activity-guided biological assay [28, 29]. The rat aorta ring assay has been used successfully to screen for compounds that modulate angiogenesis, *in vitro* [30–32]. The anti-angiogenic drug, PI-88 (Muparfostat; a sulfated oligosaccharide) [33], which has reached a phase III clinical trial in hepatocellular carcinoma patients [34], was identified using a modified version of this assay which involved human placental blood vessel fragments (Brown et al 1996).

The rat aortic ring assay is thought to provide a bridge between *in vivo* and *in vitro* angiogenesis models [32]. This assay not only includes endothelial cells but also their surrounding cells [30] and rat aortic rings cultured in collagen gel give rise to microvascular networks composed of branching endothelial channels which approximate a microvascular network. Therefore, it is thought that using the rat aorta assay reproduces more accurately the environment in which angiogenesis takes place *in vivo* than alternative procedures using preselected endothelial cells and their subsequent passaging *in vitro*. However, since angiogenesis predominantly occurs at the microvascular level, the use of large vessels, such as aorta, as the source of new vessels may be a less-than-ideal choice [30, 35]. Although the rat aorta assay is useful for screening angiogenesis modulating compounds, an understanding of the cellular mode of action requires additional bioassays to examine the individual mechanistic steps of angiogenesis, such as endothelial cell proliferation, migration, adhesion and tube formation [30].

In this paper we used a modification of the rat aorta assay [36] that enabled us to screen for, and discriminate between, pro- and anti-angiogenic activities in fractionated extracts of soybean xylem sap. Using this approach we identified three fractions of soybean xylem sap that enhanced angiogenesis and, subsequently, characterised the proangiogenic molecules in two of these fractions and determined their mode of action.

## Materials and methods

### Materials

Analytical grade reagents were obtained from Sigma-Aldrich, St. Louis, MO and Ajax Chemicals, Auburn, NSW, Australia. Soybean seeds (*Glycine max* cultivar Bragg) were obtained from Prof. Peter Gresshoff, University of Queensland, Australia. Deionised water was used in all laboratory procedures (>18.2 MΩ.cm; Millipore, Billerica, MA). Commercial *e*GGCE was purchased from BOC Sciences, New York, NY and *t*GGCE (isolated from *Bretschneidera sinensis*) was kindly provided by Prof. Dr. Wei-Dong Zhang and Dr. Shan Lei from the Second Military Medical University, Shanghai, China.

### Soybean growth and xylem sap isolation

Soybeans were grown in square pots (100 × 100 × 300 mm) containing vermiculite (Ausperl, Banksmeadow, NSW, Australia) under glasshouse conditions of 14 hr light (28°C) and 10 hr dark (25°C). Pots were watered every day with ~200 mL of urban water which was replaced with modified Herridge’s nutrients [37] containing 10 mM KNO_3_ every second day from the second week of planting.

Xylem sap, a clear solution lacking any visible signs of chlorophyll contamination, was collected from decapitated plants, 4–6 weeks post-germination [38, 39]. Sap was collected between 0–7 hr from the wound site by attaching 5 mL syringes via a short length (3–4 cm) of rubber tubing (i.d., 3 mm) fitted over the cut stump. Exudation was estimated at 0.34 mL/hr per plant over 24 hr. Sap from 50–300 plants was pooled and lyophilized overnight.

### Size fractionation and HPLC separation

Molecular weight spin filters (10 kDa and 3 kDa; Merck Millipore, Billerica, MA) were utilised in succession to remove the high molecular weight sap proteins [38] and enrich the low molecular weight sap molecules. The 3 kDa filtrate was freeze dried, redissolved in 100 μL of water (equivalent to 40 mL of fresh xylem sap) and analysed by HPLC (LC10-VP series, Shimadzu, Kyoto, Japan) using a reverse phase Alltech Platinum C18 column (5 μm, 250 × 4.6 mm) and guard cartridge (Grace Discovery Sciences, Columbia, MD). The column was held at 35°C and eluted over 30 min with a linear gradient of 5% to 85% acetonitrile (hold 5 min) at 1 mL/min. Sample components were detected with a photodiode array (PDA) detector and peaks collected by monitoring UV absorbance in real time at 254 nm. Fractions were collected from 2 to 30 min at 30 sec intervals, dried under nitrogen, diluted to 200 μL with water and either screened for activity in the pro-angiogenic bioassay or stored at -70°C.

The semi-preparative purification of ***FK1*** and ***FK2*** utilised the Alltima C18 column as above (50 μl injection, 20 mL sap equivalent) with an optimised linear gradient of 20% (hold 5 min) to 31% (over 15 min) acetonitrile with a final wash over 5 min from 31% to 85% (hold 5 min).

### Bioassay for angiogenesis modulating activity using the *in vitro* rat aorta assay

The rat aorta angiogenesis bioassay [33] was modified to enhance the ability to screen for pro-angiogenic activity in the HPLC-derived fractions by reducing the concentration of heat inactivated foetal calf serum (HIFCS) from 20% to 5% and incubating for 7 days in 48 well culture plates (Costar, Corning, Lowell, MA) at 37°C in 5% CO_2_ [36]. Six technical and three biological replicates were used and the medium was changed on day 4 of culture. Vessel outgrowth was quantified as percent growth under a Nikon TMS inverted phase contrast microscope (Nikon Instrument Inc., Tokyo, Japan) at 40 × magnification on days 5, 6 and 7. Control cultures either received medium with the diluent alone or with the known anti-angiogenic agent, PI-88 [33] added at 100 μg/mL. Vessel outgrowth was determined as the percentage of a microscope field, outside the aorta ring, that was occupied by blood vessels using ImageJ software as previously described [40] or measured manually, particularly when large numbers of column fractions needed to be scored.

### Liquid chromatography mass spectrometry (LC-MS)

Mass spectrometry of the purified pro-angiogenic components was carried out using an Agilent 6530 Accurate Mass LC-MS Q-TOF (Santa Clara, CA). Samples were subjected to electrospray ionisation (ESI) in the Jetstream interface in positive and negative modes under the following conditions: gas temperature 300°C, drying gas 4 L/min, nebulizer 35 psig, sheath gas temperature 350°C and flow rate of 11 L/min, capillary voltage 3500 V, fragmentor 175 V, and nozzle voltage 1000 V. Unfractionated xylem sap or HPLC purified pro-angiogenic components (3 μL) were injected onto an Agilent Eclipse XDB-C18 (2.1 × 50 mm column; 1.8 μm) and eluted with a linear gradient from 10 to 50% of mobile phase B in 8 min, then to 70% in 4 min (hold for 8 min) at a flow rate of 200 μL/min. Mobile phase A was water containing 0.1% formic acid; mobile phase B was acetonitrile/water (9:1 v/v) containing 0.1% formic acid. The instrument was run in extended dynamic range mode from *m*/*z* 100–3000 and data acquired by targeted collision induced dissociation (CID; N_2_ collision gas supplied at 18 psi) MS/MS (2 spectra/s). Data were acquired and analysed with Agilent’s MassHunter software.

### Nuclear magnetic resonance spectroscopy (NMR)

Nuclear magnetic resonance (NMR) spectroscopy experiments were recorded on either a Bruker AVANCE 800 or 600 MHz NMR spectrometer with TCI cryoprobe (Bruker, Billerica, MA) using D_2_O as the solvent at 298 K. Spectra were analysed using Bruker TopSpin 2.1 software. ^1^H NMR chemical shifts in parts per million (ppm) are reported using the HOD signal as an internal chemical shift reference (4.72 ppm at 298 K). ^13^C chemical shifts in ppm are referenced indirectly to the proton shift.

^***1***^***H*:** 1D proton NMR spectra were performed using a standard Bruker pulse program, *zgpr*, which included solvent suppression via pre-saturation. A total experiment time of 5 min was used with a *t*_1max_ of 1.25 sec. ^***1***^***H-***^***1***^***H DQF-COSY*:** A phase-sensitive DQF-COSY spectrum was also measured using the standard Bruker sequence *cosydfphpr*, which includes a double-quantum filter and pre-saturation. A total experiment time of 38 min was used with a *t*_1max_ of 26 msec and a *t*_2max_ of 104 msec. ^***1***^***H-***^***1***^***H TOCSY*:** A TOCSY spectrum was measured using the Bruker pulse program *mlevphpp* that was modified to include pre-saturation. A total experiment time of 19 min was used with a *t*_1max_ of 13 msec, a *t*_2max_ of 250 msec and a TOCSY mixing time of 60 msec. ^***1***^***H-***^***1***^***H NOESY*:** A NOESY spectrum was recorded using the Bruker pulse program *noesygpphpp* modified to include pre-saturation. A total experiment time of 14.2 hr was used with a *t*_1max_ of 39 msec, a *t*_2max_ of 1.62 sec and a 500 msec NOE mixing time. ^***13***^***C-***^***1***^***H HSQC*:** A ^13^C-HSQC spectrum was recorded using the standard Bruker pulse sequence *hsqcedetgpsisp2*.*2*. A *t*_1max_ of 15.3 msec, a *t*_2max_ of 152 msec and a total experiment time of 2.3 hr was used. ^***13***^***C-***^***1***^***H HMBC*:** A ^13^C-HMBC spectrum was recorded using the standard Bruker pulse sequence *hmbcetgpl2nd*, which includes a two-fold low-pass *J*-filter to suppress one-bond correlations. A *t*_1max_ of 10.1 msec, a *t*_2max_ of 304 msec and a total experiment time of 8 hr was used.

### Cell proliferation (mitogenic) assay

Human umbilical vein endothelial cell (HUVEC) proliferation was assessed by determining ^3^H-thymidine incorporation [41]. HUVECs were cultured in M199 medium supplemented with 20% HIFCS, 0.24 mg/mL gentamycin, 2 mM L-glutamine, 0.04 mg/mL endothelial cell growth supplement (ECGS) (Sigma-Aldrich, St. Louis, MO) and 0.135 mg/mL heparin (Sigma-Aldrich, St. Louis, MO) in gelatin-coated 96-well plates and incubated for 4 days at 37°C in 5% CO_2_ to reach confluence. The confluent cells were then serum starved for 24 hr. Subsequently, 100 μL/well of test compound in serum-free medium with or without basic fibroblast growth factor (bFGF) (R&D Systems, Minneapolis, MN) was added to each well for another 24 hr before adding ^3^H-thymidine. ^3^H-thymidine (0.5 μCi/well; MP Biomedicals, Solon, OH) was added and incubated for the final 24 hr. Incubation was stopped by adding 100 μL/well of trypsin/EDTA to lift the cells from the gelatin layer with 100 μL/well of M199 medium supplemented with 20% HIFCS added to neutralize the trypsin enzymatic activity. Finally, plates were frozen and thawed 3 times and cells were harvested using a 96-well cell Filtermate 196 harvester, with EasyTab^TM^-C self-aligning filters and the incorporated radioactivity counted with a Topcount^®^NXT™ Microplate Scintillation and Luminescence Counter (Packard Bioscience, Meriden, CT).

### Cell migration assay (wound healing assay)

Label-free, kinetic assays for cell migration used the IncuCyte^TM^ live-cell imaging system (Essen BioSciencen, Ann Arbor, MI) following the techniques described previously [42]. Briefly, human microvascular endothelial cells (HMEC) were added to 96-well ImageLock Essen plates (Essen BioSciences, Ann Arbor, MI) at 2.5 × 10^4^ cells/well in 100 μL/well of MCDB 131 medium (Invitrogen) supplemented with 10% HIFCS, 0.1% PSN (0.03 g/L penicillin G, 0.05 g/L streptomycin sulfate, 0.05 g/L neomycin sulfate), 2 mM L-glutamine, 0.01 μg/mL endothelial cell growth factor (ECGF), (Gibco BRL, Grand Island, NY) and 1 μg/mL hydrocortisone (Sigma-Aldrich, St. Louis, MO) and incubated for 2 days at 37°C in 5% CO_2_ to reach confluence. After removing the medium from each well, a wound was made in the HMEC monolayer using the 96-pin WoundMaker device. The use of Essen ImageLock plates ensured that the wounds were automatically located and registered by the IncuCyte software for imaging and data recording.

Each well was then rinsed twice with 100 μL/well of culture medium to prevent dislodged cells from setting and re-attaching. Medium (100 μL/well), with or without test compound, was added to each well. Wound images were then captured and saved at 2 hr intervals until control wounds had recovered completely. The data was analysed either by the IncuCyte software package or extracted by three integrated metrics (wound width, wound confluence, relative wound density) and analysed by Prism statistical software.

### Endothelial cell tube formation assay

HMEC and HUVEC cultures were used for *in vitro* endothelial tube formation. Matrigel (BD Biosciences, Bedford, MA) was thawed overnight at 4°C and plated into ice-cold 96-well ImageLock Essen plates at 50 μL/well using pre-cooled pipets, tips and tubes. The plates were incubated at 37°C in 5% CO_2_ for 1 hr to allow the Matrigel to form a stable gel. HUVECs at 4×10^4^ cells/well in 100 μL of M199 medium supplemented with 20% HIFCS, 0.24 mg/mL gentamycin, 2 mM L-glutamine, 0.04 mg/mL ECGS, and 0.135 mg/mL heparin with or without test compounds were added and Matrigel cultures placed inside the IncuCyte and incubated at 37°C for 24 hr.

HMEC (100 μL; 5×10^4^ cells/well) in MCDB 131 medium supplemented with 10% HIFCS, 0.1% PSN, 2 mM L-glutamine, 0.01 μg/mL ECGF, and 1 μg/mL hydrocortisone ± test compounds were added to the Matrigel. Tube formation was imaged at 2 hr intervals using the IncuCyte^TM^ live-cell imaging system with the phase-contrast ImageLock scan type. The images were analysed by measuring percentage of denuded area and number of sprouting cells at early stages of tube formation (4 hr) and the total number and length of tubes formed at later stages of tube formation (6 hr) in each well using IncuCyte and NIH ImageJ software with the Angiogenesis Analyzer plugin [43] for quantification of tube networks.

### Rose Bengal cell adhesion assay

The Rose Bengal cell adhesion assay is based on a previously described method [44] used to measure the interaction of antibodies with cell surface antigens using an automated colorimetric assay. A 96 round bottom (U-well) culture plate (Costar, Corning, Lowell, MA) was first coated either with bovine plasma fibronectin (Invitrogen, Carlsbad, CA) or purified human vitronectin (Gibco BRL, Grand Island, NY) at 50 μL/well at selected concentrations ranging from 0.313 to 10 μg/mL and incubated overnight at 4°C. The liquid layer was then removed and the cells washed by submerging twice in a PBS bath (removing the PBS between washes). The non-specific binding sites were then blocked by incubation with 200 μL/well of 1% BSA in Hank’s balanced salt solution (HBSS) for 1 hr at 37°C before removing the supernatant. HMEC (5 × 10^4^ cells/well) were added in 100 μL/well of serum free culture medium supplemented with 0.1% BSA with or without the test compounds. The plate was then incubated for (2.5–60 min) at 37°C. Unbound cells were removed and 100 μL/well of 0.25% (w/v) Rose Bengal dye (Koch-Light Laboratories Ltd., Colnbrook, Berkshire, England) in PBS was added to stain the bound cells, for 3 min at room temperature. The non-absorbed dye was then removed and the plate washed twice by submerging in fresh PBS baths.

After draining the plate, 200 μL/well of 50% ethanol in PBS was added and mixed to liberate dye from the cells. Non-specific binding of the dye to fibronectin/vitronectin-coated and uncoated wells in the presence and absence of cells was also determined and subtracted. The relative number of bound-cells in each well was quantified by determining each well’s optical density (OD) at λ_1_ = 540 nm and λ_2_ = 650 nm, on a Thermomax microplate reader (Molecular Devices, Sunnyvale, CA). The data was then analysed by GraphPad prism 5.04 software (GraphPad Software, San Diego, CA).

### Statistical analyses

Data are reported as mean ± SEM. Statistical significance was measured using a two-tailed unpaired *t-*test between the sample treated and untreated groups and one-way and two-way ANOVA, comparing each group to control and other groups using the GraphPad prism 5.04 software. *P* values less than 0.05 were considered statistically significant.

## Results

### Isolation and fractionation of low molecular weight pro-angiogenic molecules from soybean xylem sap

**Fig 1:**
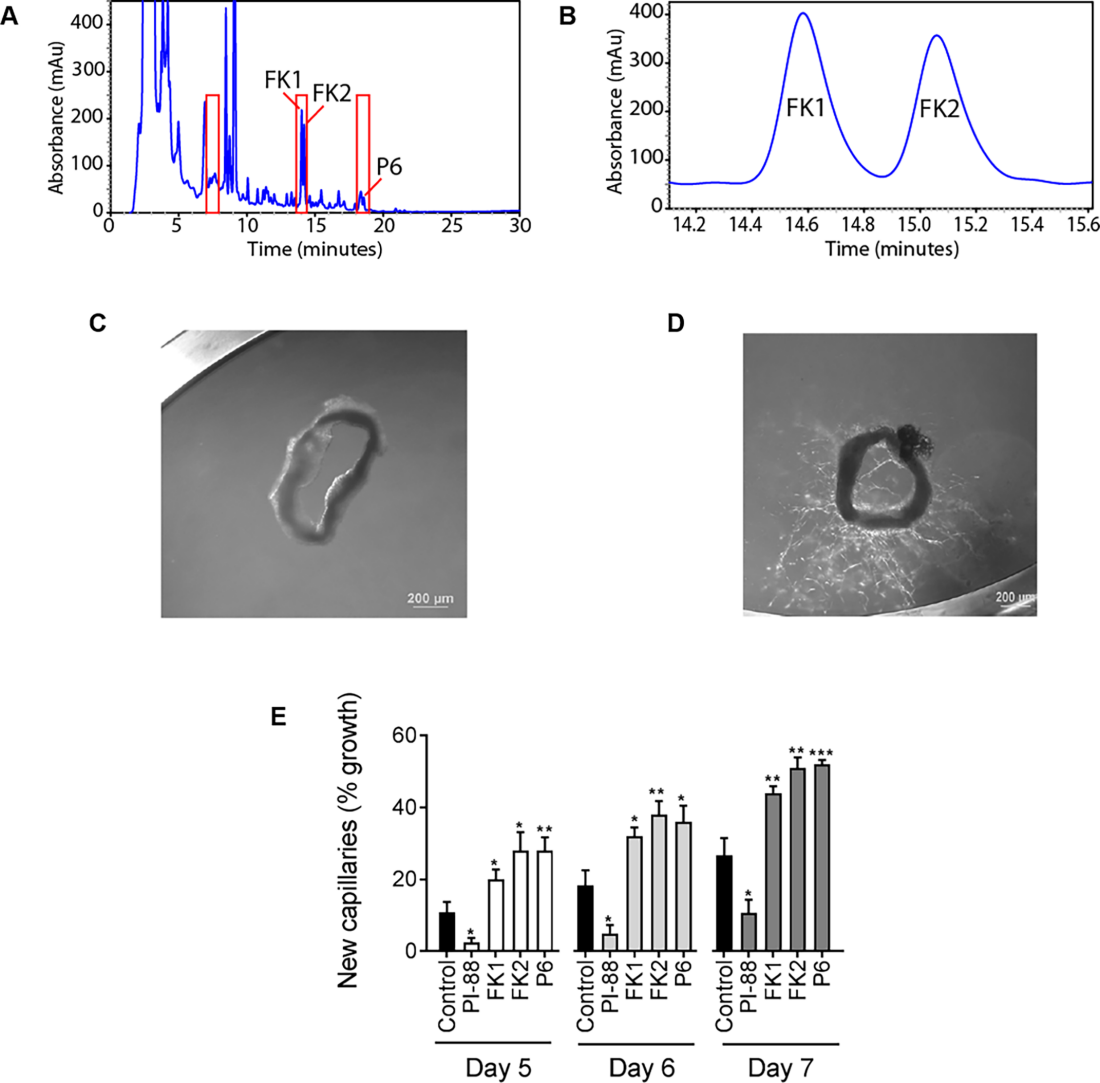
Biological activity of HPLC fractionated soybean xylem sap. A, UV absorbance at 254 nm of HPLC separated xylem sap material, the injection volume of 50 μL containing a sap concentrate equivalent to 20 mL of sap. Fractions were collected at 30 s intervals and tested by the rat aorta ring assay and fractions with pro-angiogenic activity are highlighted in red. B, Separation of ***FK1*** and ***FK2*** using a shallow gradient for a large scale purification (see methods). C, D The pro-angiogenic activity of the test compounds is expressed as a percentage of aorta vessel outgrowths observed on days 5, 6 and 7 based on the field of view, with (C) depicting 0% outgrowth and (D) 60% outgrowth. E, Biological activity of fractions containing purified ***FK1***, ***FK2*** or ***P6*** using the rat aorta ring assay. Day 5–7 results are shown. Control cultures received medium with the diluent only or diluent with the anti-angiogenic compound, PI-88, at 100 μg/ml.

Concentrated soybean xylem sap was fractionated using reversed-phase C18 HPLC chromatography and screened for the presence of hydrophobic, low molecular weight angiogenesis-modulating molecules using the rat aorta ring bioassay. Although yield varied depending upon plant growth, fractions eluting at 7–7.5, 13.5–14 and 18–18.5 min gave highly consistent and significant pro-angiogenic activity when measured at days 5, 6 and 7 using the rat aorta assay (Fig 1A). We focussed on the more hydrophobic fractions at 13.5–14 and 18–18.5 min because activity strongly correlated with the presence of UV absorbing peaks at these elution times (S1 Fig). The UV absorbing and biologically active fractions were separated using an optimised HPLC gradient (Fig 1B). Two peaks eluting between 13.5–14 min gave identical UV absorption spectra each of which were distinguishable from the peak at 18–18.5 min (S1 Fig). The three purified fractions (designated ***FK1***, ***FK 2*** and ***P6***) showed significant pro-angiogenic activity (Fig 1C–1E). Upon long term storage the activity of ***P6*** diminished, suggesting instability, but ***FK1*** and ***FK2*** were stable. Due to instability and insufficient material, ***P6*** was not analysed further.

### NMR analysis of purified *FK1* and *FK2*

A scaled-up extraction of 300 plants yielded 2000 mL of xylem sap. Optimization of the HPLC separation of ***FK1*** and ***FK2*** enabled the isolation of 458 μg of ***FK1*** and 387 μg of ***FK2*** of sufficient purity to enable NMR and MS analysis. The pro-angiogenic activity of the purified ***FK1*** and ***FK2*** was confirmed by bioassay before nuclear magnetic resonance (NMR) and mass spectrometry (MS) analysis (see below) which validated that the isolated ***FK1*** and ***FK2*** were pure, UV absorbing and biologically active.

Initial 1D ^1^H NMR spectra were recorded for both ***FK1*** and ***FK2*** products. ***FK1*** was determined to be more pure and of higher concentration, so initial structure determination was performed on this product. ^13^C-HSQC, ^13^C-HMBC, ^1^H-^1^H DQF-COSY, ^1^H-^1^H TOCSY and ^1^H-^1^H NOESY NMR spectra were then measured of ***FK1*** and structural fragments were constructed as follows. Two methoxy resonances were identified in the ^1^H NMR spectrum at δ 3.68 (3H, s, **H-10**) and δ 3.70 (3H, s, **H-10’**). Two protons at δ 6.56 (1H, dt, *J* = 16.0, 1.0 Hz, **H-8’**) and δ 6.30 (1H, dt, *J* = 15.9, 6.0 Hz, **H-7’**) were identified in a *trans* double bond arrangement due to their ^3^*J* coupling of 16.0 Hz, and were linked to a degenerate methylene group at δ 4.25 (2H, dd, *J* = 6.0, 0.9 Hz, **H**_**2**_**-9’**) via their ^3^*J* and ^4^*J* couplings, respectively. Proton signals at δ 4.07 (dd, ^2^*J* = 12.3 Hz, ^3^*J* = 2.8 Hz, 1H, **H-9a**) and δ 3.94 (dd, ^2^*J* = 12.3 Hz, ^3^*J* = 6.7 Hz, 1H, **H-9b**) were linked through their ^2^*J* coupling and their shared carbon shift in the ^13^C-HSQC spectrum, and were linked to a multiplet at δ 4.66 (m, 1H, **H-8**) via the DQF-COSY. An ABX aromatic coupling system was identified, and positioned the aromatic proton at δ 6.86 (dd, ^3^*J* = 8.1 Hz, ^4^*J* = 1.9 Hz, 1H, **H-6**) *meta* from δ 6.88 (d, ^4^*J* = 2.0 Hz, 1H, **H-2**) and *ortho* to δ 6.74 (d, ^3^*J* = 8.1 Hz, 1H, **H-5**). Three further aromatic protons were identified and linked via the TOCSY cross-peaks, but needed correlations from the HSQC and HMBC spectra to resolve δ 6.97, (overlapped s, 1H, **H-6’**) as *ortho* to δ 6.97, (overlapped s, 1H, **H-5’**), and *para* to δ 6.98 (bs, 1H, **H-2’**). The HSQC and HMBC spectra were also used to resolve a solvent overlapped peak, Hδ 4.7 Cδ 72.54 (overlapped m, 2H, **H-7**, **C-7**) and link this resonance to Cδ 61.52 (**C-9**). The methoxy group **H**_**3**_**-10** was linked to **H-2** via the NOESY spectrum and to Cδ 147.72 (**C-3**) via the HMBC spectrum, which was also linked to **H-6** and **H**-**5**. Methoxy **H**_**3**_**-10** was linked to **H-2’** via the NOESY spectrum and to Cδ 149.22 (**C-3’**) via the HMBC spectrum, which was further linked to **H-5’** and **H-6’**. NOEs were observed between **H-2’**, **H-5’** and **H6’** to **H-7’** and **H-8’**, linking these fragments, and was positioned by the HMBC correlation from **H-7’** to Cδ 119.45 (**C-6’**) and Cδ 109.78 (**C-2’**) as was **H-8’** to Cδ 131.13 (**C-1’**). Finally, HMBC correlations between **6-H**, **2-H** and **C-7** and further correlations between **H-8** and Cδ 146.94 (**C-4’**) constructed the final compound.

**Fig 2:**
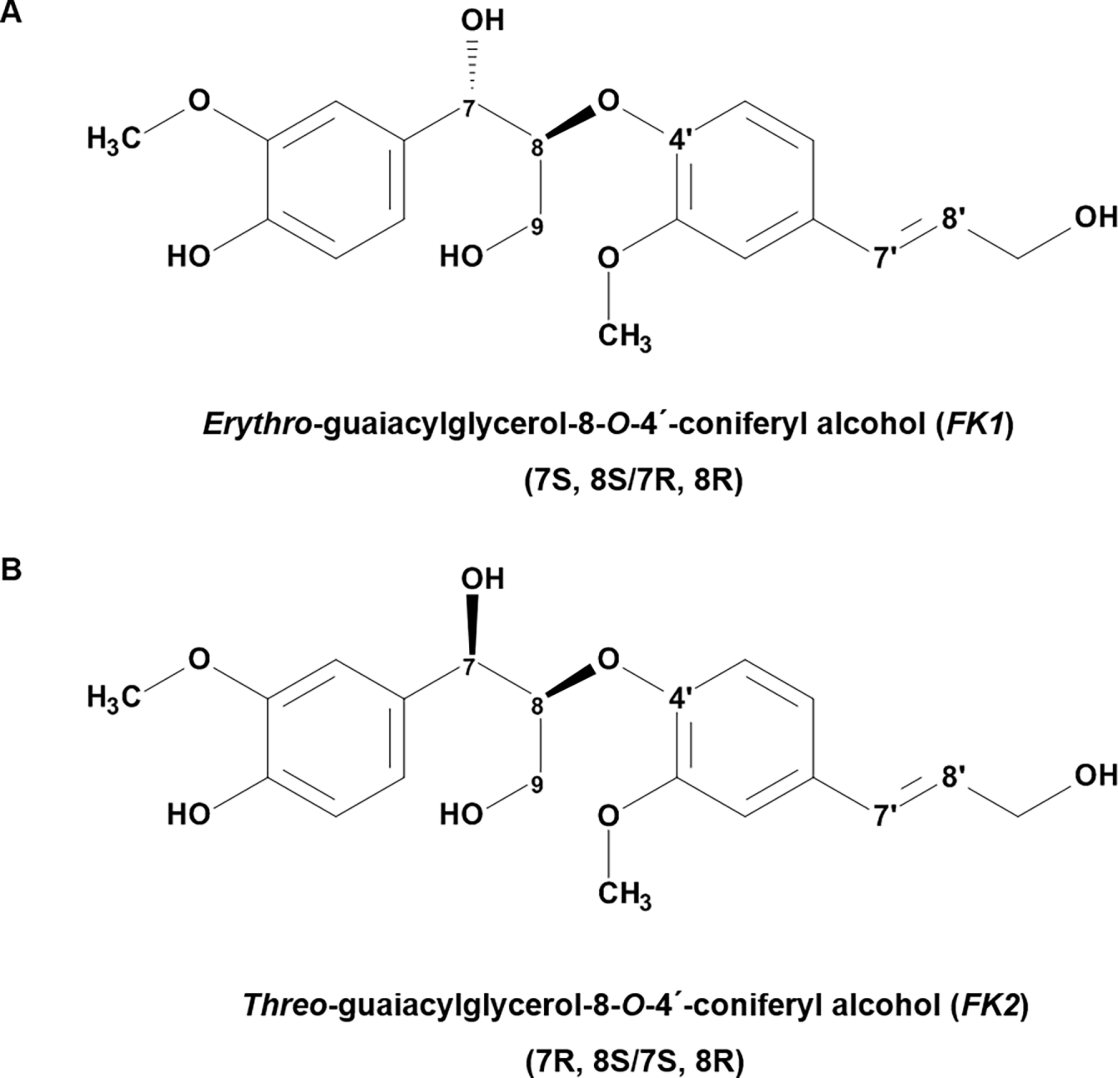
Chemical structures of purified *FK1* and *FK2*. Chemical structures of ***FK1*** (*e*GGCE) (**A**) and ***FK2*** (*t*GGCE) (**B**), which are coniferyl neolignans, based on the NMR, MS and UV spectroscopic data described in this paper, with *e*GGCE and *t*GGCE containing two chiral carbons (C-7 and C-8). The NMR, MS, UV spectroscopic and pro-angiogenic properties of the isolated ***FK1*** and ***FK2*** were also comparable to commercial *e*GGCE purchased from BOC Sciences, NY, USA, and to naturally-derived *t*GGCE provided by the Second Military Medical University, Shanghai, China.

Assignments could easily be made for ***FK2*** due to the similarity of the ^13^C-HSQC cross-peaks to those of ***FK1*** and ^1^H-^1^H couplings constants (S2 Fig). It was then proposed that ***FK1*** and ***FK2*** were diastereomers, with undefined chirality of **C-7** and **C-8**. The compound identified (Fig 2) was proposed to be the previously described natural product guaiacylglycerol-8-*O*-4’(coniferyl alcohol) ether (GGCE), coniferyl neolignans of which there are four possible stereoisomers. The NMR spectra were found to be in agreement with previously reported chemical shifts and couplings [45].

### LC-MS/MS analysis of purified *FK1* and *FK2*

**Fig 3:**
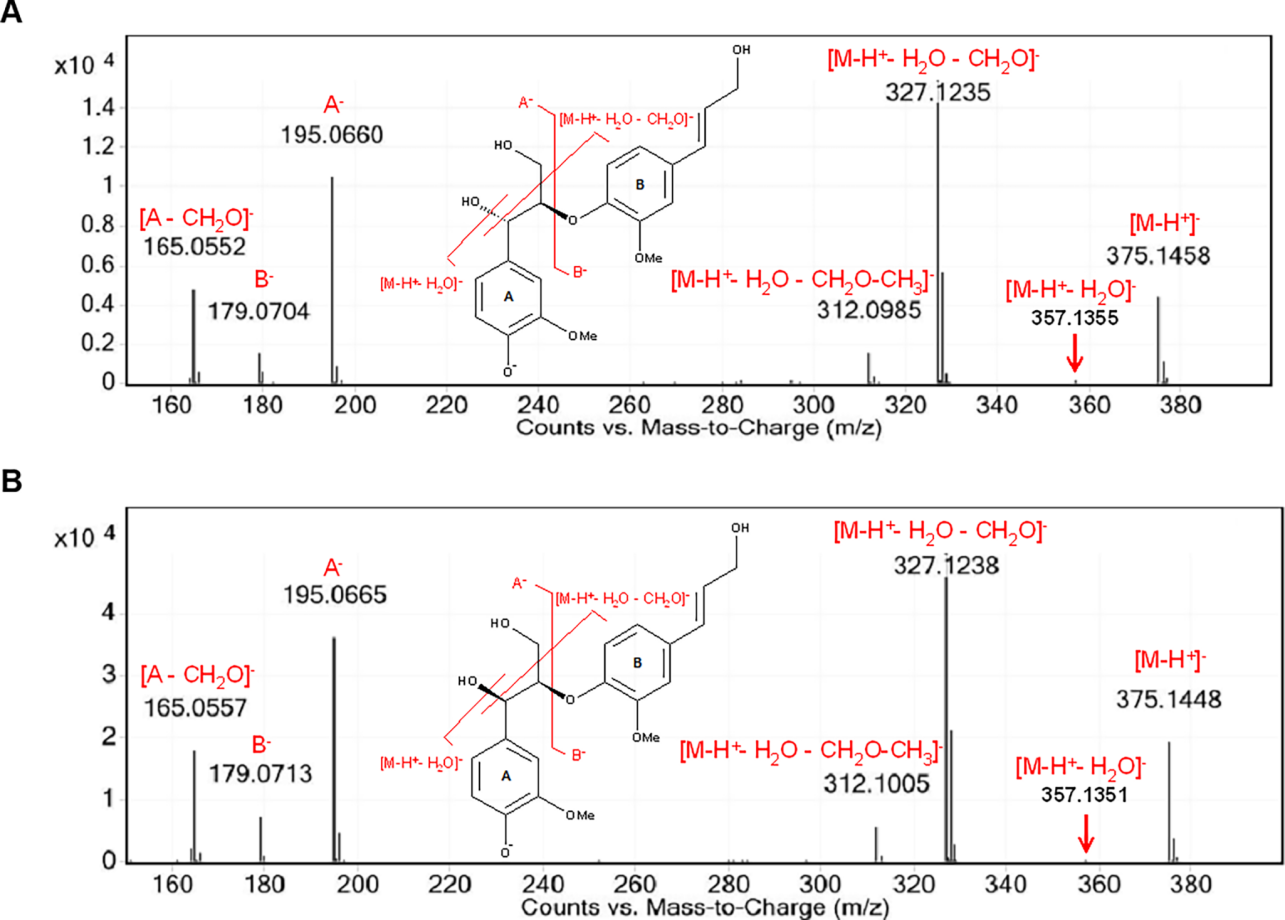
Negative ion mode ESI-MS/MS spectra analysis of *FK1* and *FK2*. ***FK1***, *erythro*-guaiacylglycerol-8-*O*-4´-(coniferyl alcohol) ether (*e*GGCE) (A) and ***FK2***, *threo*-guaiacylglycerol-8-*O*-4´-(coniferyl alcohol) ether (*t*GGCE) (B) spectra, selecting *m/z* 375 ([M-H]^-^) for CID at 10 eV.

Initial QTOF MS data implied a chemical formula of C_20_H_22_O_6_, for both ***FK1*** and ***FK2***. However, through the NMR structure elucidation process, an extra proton shift was identified correlated to the spin system of ***FK1***. It was proposed that the mass initially observed in the QTOF MS was in fact the [M-H_2_O]^-^ peak, implying a formula of C_20_H_24_O_7_. This was confirmed by reducing the nebulizer spray temperature to reveal the predicted molecular ions for ***FK1*** and ***FK2*** by both -ve ESI ([M-H]^-^, *m/z* 375) (Fig 3 and S1 Table) and +ve ESI (M^+^, *m/z* 376; and [M+Na]^+^, *m/z* 399) (S3 Fig and S2 Table).

The deprotonated ***FK1*** and ***FK2*** molecules (*m/z* 375, [M-H]^-^) were essentially identical to those reported by Morreel *et al*. [46], comprising a series of losses of H_2_O (*m/z* 357, [M-H-H_2_O]^-^), formaldehyde (*m/z* 327, [M-H-H_2_O-CH_2_O]^-^) and a methyl moiety (*m/z* 312, [M-H-H_2_O-CH_2_O-CH_3_]^-^) (Fig 3) [46, 47]. A comparison of the observed and calculated masses for the two deprotonated isomers and their associated fragments showed them to be in good agreement (Δppm between -3.19 and 4.85) (S2 Table).

In the positive mode, the MS spectra was dominated by the sodium adduct (*m/z* 399 ([M+Na]^+^) and successive losses of OH (*m/z* 359, [M-OH]^-^), H_2_O (*m/z* 341, [M-OH- H_2_O]^-^) and formaldehyde (*m/z* 311, [M-OH- H_2_O-CH_2_O]^-^) (S3 Fig). A comparison of the observed and calculated masses for these ions showed them to also be in good agreement (Δppm between -1.32 and 0.58) (S2 Table). It is worth noting the presence of the odd electron ion [M]^+^ (S3 Fig). Although it was present in low abundance, this was still sufficient to calculate an elemental formula from the measured accurate mass. Odd electron molecular ions are occasionally observed in ESI MS, particularly when compounds with low redox potential are analysed at low flow rates so that the neutral species is in contact with the capillary/solution interface for a longer time [48].

The NMR and MS data are all consistent with ***FK1*** and ***FK2*** being identified as diastereomers of GGCE, namely *erythro-*guaiacylglycerol-8-*O*-4´-(coniferyl alcohol) ether (*e*GGCE) and *threo-* guaiacylglycerol-8-*O*-4´-(coniferyl alcohol) ether (*t*GGCE) (Fig 2), which are classified as neolignans.

In addition, a commercially purchased sample of *e*GGCE and an independently derived natural product isolate of *t*GGCE (isolated from *Bretschneidera sinensis*) were sourced and found to have near identical spectroscopic and chromatographic properties as the isolated ***FK1*** and ***FK2***, respectively (data not shown).

### *In vitro* activity of independently sourced *e*GGCE (*FK1*) and *t*GGCE (*FK2*) in the rat aorta ring bioassay

**Fig 4:**
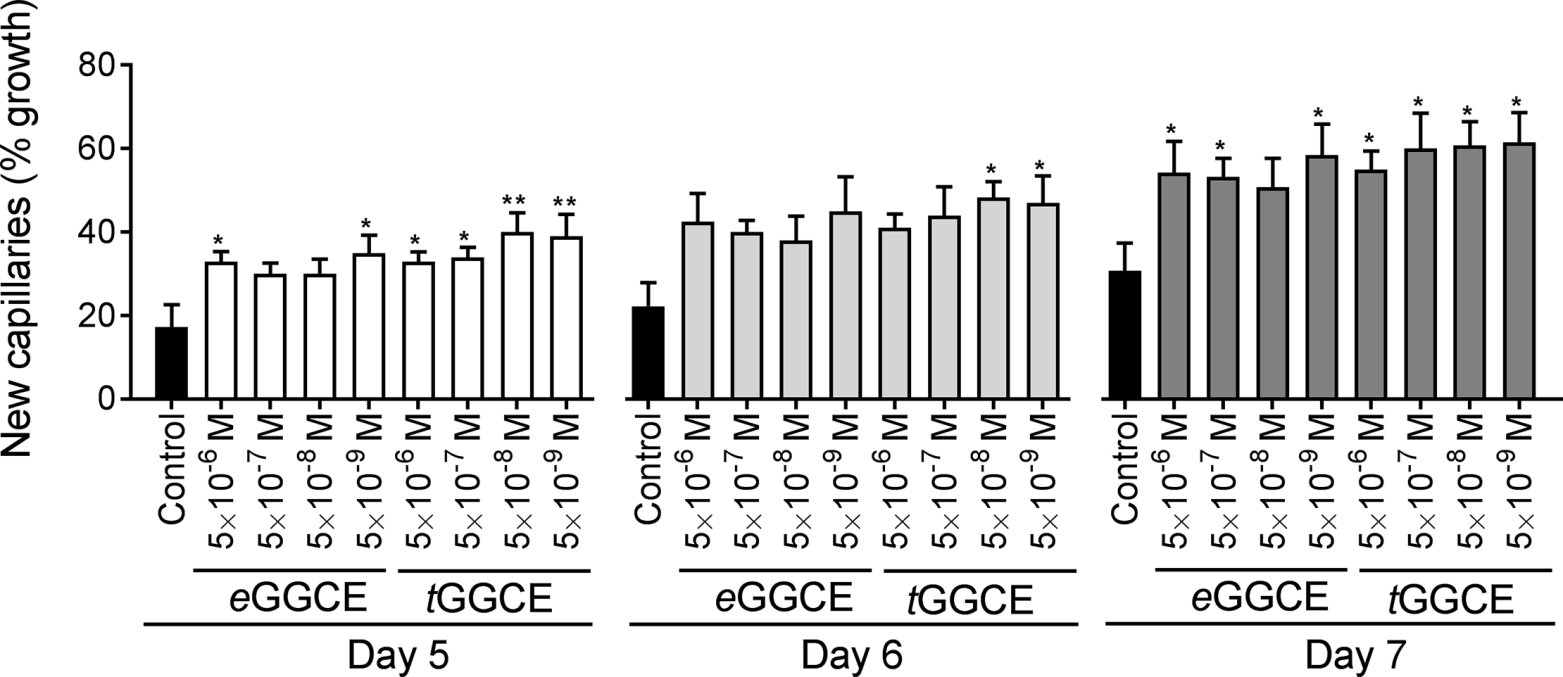
Pro-angiogenic activity of *e*GGCE *(FK1)* and *t*GGCE (*FK2*) in the *in vitro* rat aorta assay. The independently sourced *e*GGCE and *t*GGCE preparations were tested at concentrations ranging from 5 × 10^−6^ M to 5 × 10^−9^ M. Control cultures contained the same diluent dilution as the test compounds. Data are expressed as a percentage of aorta vessel outgrowths observed on days 5, 6 and 7. Data analysis was performed by Student–Newman–Keuls test after one-way ANOVA comparing each group to control in each treatment. Error bars represent SEM (n = 6). *, P ≤ 0.05, **, ≤ 0.01.

The biological activity of the independently sourced *e*GGCE and *t*GGCE were investigated over a range of concentrations (5 × 10^−6^ M to 5 × 10^−9^ M) to confirm that they had the same activity as those derived from soybeanThe results, expressed as percent growth, showed significant enhancement of vessel outgrowth by both *e*GGCE and *t*GGCE compared to the control after 5, 6 and 7 days of culture at the concentrations examined (Fig 4).

### Effects of *e*GGCE (*FK1*) and *t*GGCE (*FK2*) on endothelial cell proliferation

**Fig 5:**
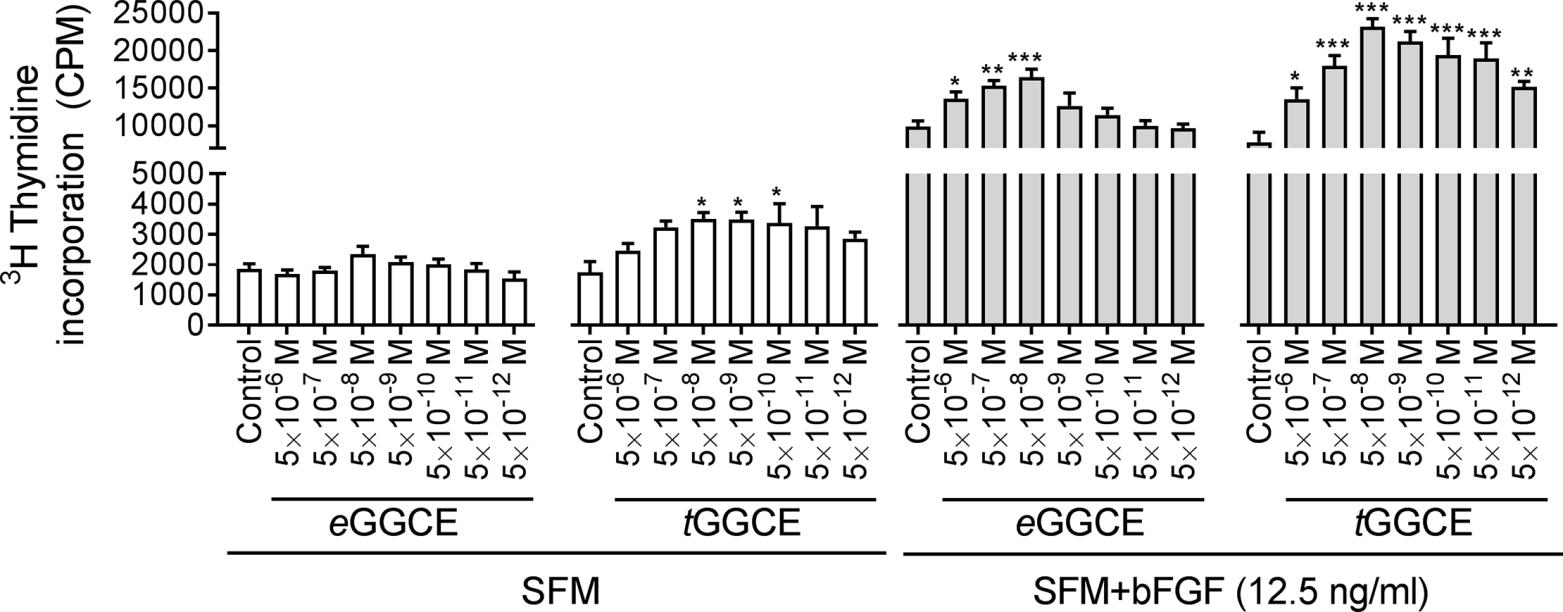
HUVEC proliferation assay in the presence of eGGCE *(FK1)* and tGGCE *(FK2)*. Effect of *e*GGCE and *t*GGCE, at concentrations from 5 × 10^−6^ M to 5 × 10^−12^ M on confluent serum-starved HUVEC proliferation in serum free media (SFM) with and without the mitogen bFGF (12.5 ng/mL). All the treatments were undertaken in the same experiment and control cultures contained the same diluent dilution as the test compounds. Cell proliferation was measured as ^3^H thymidine incorporation after 24 hr incubation. Data analysis was performed by Student–Newman–Keuls test after one-way ANOVA comparing each group to control in each treatment. Error bars represent SEM (n = 6). *, P ≤ 0.05, **, ≤ 0.01, ***, ≤ 0.001. Also, a two-way ANOVA data analysis showed a significant difference for all tested concentrations comparing their effect in SFM to SFM+bFGF (12.5 ng/mL) treatments, P ≤0.0001.

*e*GGCE and *t*GGCE were tested at concentrations ranging from 5 × 10^−6^ M to 5 × 10^−12^ M for their effect on the proliferation of serum-starved confluent HUVEC with or without the mitogen bFGF (at 12.5 ng/mL). In the presence of bFGF, these molecules significantly induced HUVEC proliferation at 5 × 10^−6^ M to 5 × 10^−8^ M and 5 × 10^−6^ M to 5 × 10^−12^ M concentrations, respectively (Fig 5). The peak of activity occurred at 5 × 10^−8^ M in both cases and tapered off at lower and higher concentrations, with *t*GGCE being more active than *e*GGCE at the lower concentrations (i.e., 5 × 10^−9^ M to 5 × 10^−12^ M). In addition, the results showed that in the absence of bFGF, *t*GGCE (but not *e*GGCE) significantly enhanced HUVEC proliferation between 5 × 10^−8^ M to 5 × 10^−10^ M compared to the control. Based on these findings it can be concluded that both neolignans *e*GGCE and *t*GGCE can enhance endothelial proliferation, but *t*GGCE is more effective than *e*GGCE.

### Effect of *e*GGCE (*FK1*) on endothelial cell differentiation (tube formation assay)

**Fig 6:**
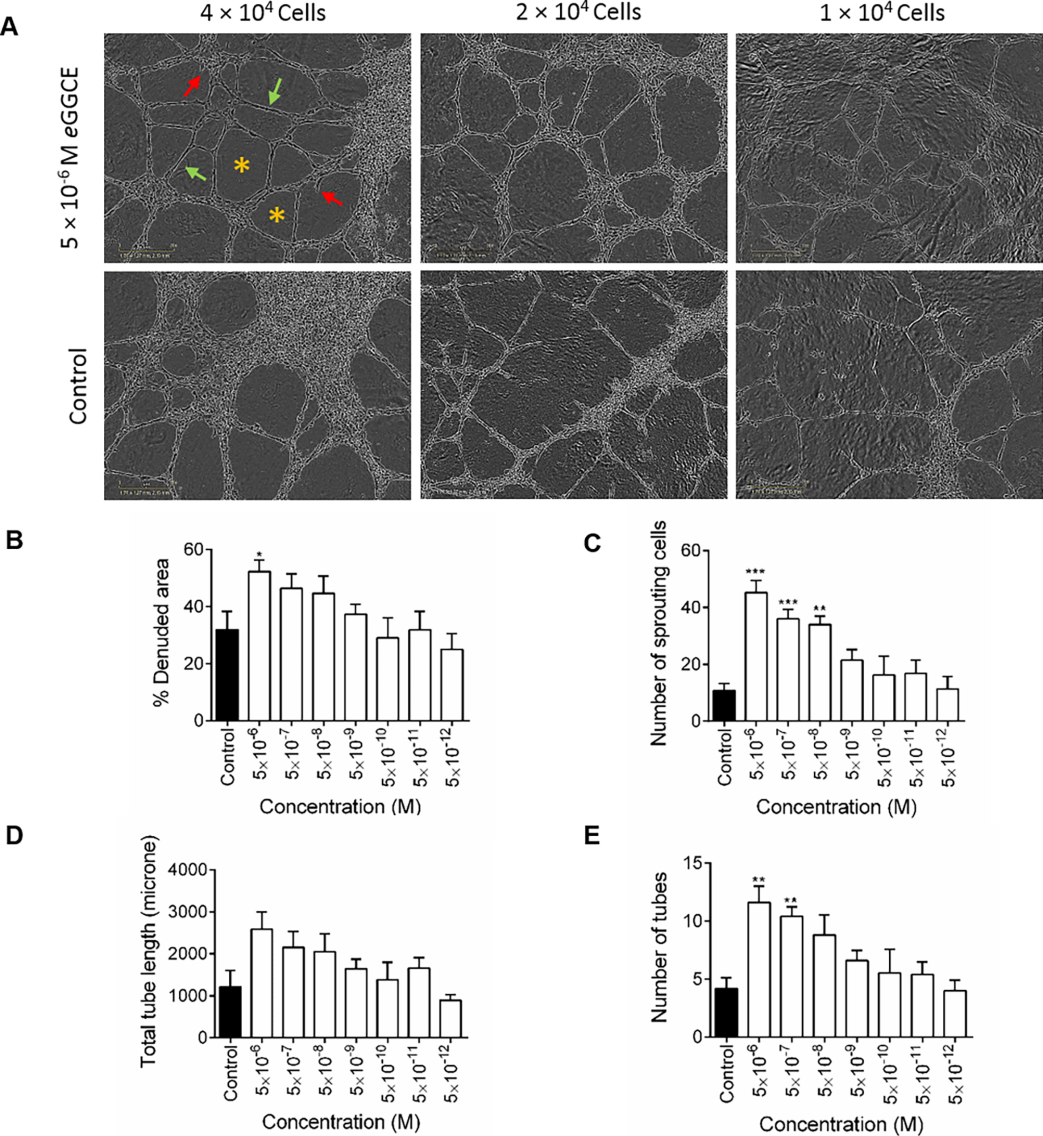
Effect of eGGCE *(FK1)* on various parameters of a HUVEC tube formation assay on Matrigel. (A) Microscopic view of isolated endothelial cells ranging from 4 × 10^4^ to 1 × 10^4^ cells/well cultured for 6 hr on Matrigel in the absence of compound, or following addition of 5 × 10^−6^ M *e*GGCE which enhanced tube formation. (B-E) Effect of *e*GGCE, at concentrations from 5 × 10^−6^ M to 5 × 10^−12^ M, on HUVEC tube formation (4 × 10^4^ cells/well) as measured by (*, B) percentage denuded area, (→, C) number of sprouting cells, (→, D) total tube length and (E) number of tubes. Parameters shown in (B) and (C) were measured after 4 hr culture and in (D) and (E) after 6 hr culture. Control cultures contained the same diluent dilution as the test compounds. Data analysis was performed by Student–Newman–Keuls test after one-way ANOVA comparing each group to control in each treatment. Error bars represent SEM (n = 6). **, ≤ 0.01, ***, ≤ 0.001.

HUVEC and HMEC tube formation assays were conducted on Matrigel in the presence and absence of *e*GGCE and the number of tubes, the number of branch points, the length of tubes, and the percentage of area covered by tubes versus total area were assessed (Aranda and Owen, 2009) using NIH ImageJ [43] and IncuCyte software. Initially, different concentrations of HUVEC (1–4 × 10^4^/well) were cultured for 6 hr with or without 5 × 10^−6^ M *e*GGCE, with enhanced tube formation by HUVECs being observed at all three cell concentrations tested (Fig 6A). Subsequent studies were undertaken at the highest HUVEC concentration (4 × 10^4^/well) and *e*GGCE was shown to enhance HUVEC tube formation above control levels with all four measured parameters, with the enhancing activity tapering down with increasing dilution (Fig 6B–6E). Denuded areas and total tube length were enhanced only at 5 × 10^−6^ M, the highest concentration tested, this enhancement being significant for denuded area, whereas the total number of sprouting cells and the number of tubes was significantly higher with *e*GGCE addition at the three and two highest concentrations tested, respectively (5 × 10^−6^ M to 5 × 10^-8^M) (Fig 6B–6E). With HMEC (4 × 10^4^ cells/well), tube formation was best measured by circle formation and was evident after 2 hr incubation, with an enhanced response being observed at the two highest *e*GGCE concentrations tested (5 × 10^−6^ M and 5 × 10^−7^ M) (S4 Fig). Based on these data it can be concluded that the neolignan, *e*GGCE, can enhance tube formation by human endothelial cells from two different sources, namely the umbilical vein and the microvasculature.

### Effect of *e*GGCE (*FK1*) on endothelial cell migration (wound healing assay) and endothelial cell adhesion (Rose Bengal assay)

An endothelial cell migration assay based on cell migration into a denuded area was employed using HMEC to determine the effect of a range of *e*GGCE concentrations (5 × 10^−6^–5 × 10^−12^ M) on endothelial cell migration. The extent and speed of wound closure was monitored microscopically every 2 hr over 26 hr (S5 Fig). In six independent experiments (n = 6), *e*GGCE imparted no measurable effect on the speed and extent of wound closure (S6 Fig).

**Fig 7:**
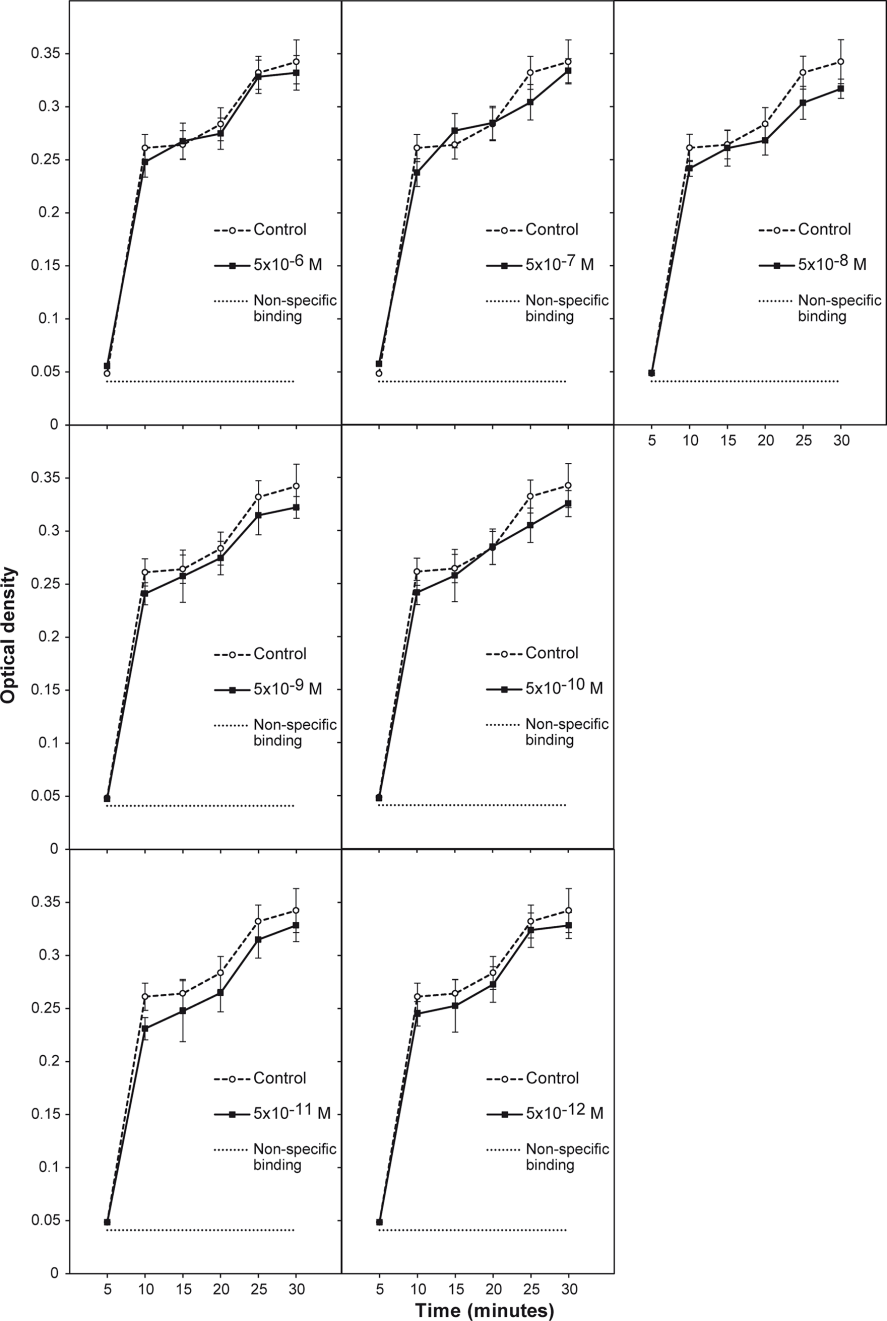
Time course of the effect of *e*GGCE (*FK1*) on fibronectin-mediated HMEC cell adhesion. Effect of *e*GGCE on cell adhesion, at concentrations ranging from 5 × 10^−6^ M to 5 × 10^−12^ M, was measured using wells coated with 10 μg/mL of fibronection and after 5–30 min incubation as optical density of Rose Bengal staining adherent cells. Data analysis was performed by one-way and two-way ANOVA, comparing each group to control and other groups. Error bars represent SEM (n = 6). No significant difference in cell adhesion was observed.

Since *e*GGCE was able to significantly enhance HUVEC and HMEC tube formation on the artificial basement membrane, Matrigel, we examined the effect of this compound on cell adhesion to the ECM components, fibronectin and vitronectin, using a Rose Bengal adhesion assay with HMEC. The fibronectin and vitronectin concentrations required for cell adhesion were first optimised by coating the microplate plastic wells with a concentration range of fibronectin and vitronectin (0 to 10 μg/mL) to establish the concentration where sub-optimal cell binding was observed. Under these conditions it was reasoned that both enhancement and inhibition of cell adhesion may be detected in the presence of *e*GGCE at different concentrations. The results showed the optimal concentrations of fibronectin and vitronectin for cell binding were 10 μg/mL and 5 μg/mL, respectively, and sub-optimal cell binding occurred at concentrations ranging from 0.313 to 2.5 μg/mL (S7 Fig). *e*GGCE did not significantly affect HMEC adhesion to fibronectin or vitronectin after 30 or 60 min adhesion nor over a time course from 5–30 min (Fig 7 and S8 Fig). Thus, it was concluded that *e*GGCE had no significant effect on either endothelial cell migration or endothelial cell adhesion to the ECM components fibronectin and vitronectin.

## Discussion

An activity-guided rat aorta ring bioassay with a proven ability to identify anti-angiogenic compounds [33] was modified by reducing the concentration of HIFCS from 20% to 5% [36]. This slowed the rate of tube formation but enabled the screening of pro- and anti-angiogenic activities over three consecutive days. Using this assay in combination with size fractionation and HPLC, soybean xylem sap derived from plants grown under several conditions was repeatedly shown to have three fractions of pro-angiogenic activity. The complexity of the UV spectrum for the earliest eluting material combined with its hydrophilic nature and the instability of ***P6***, led us to focus on fractions which contained the UV absorbing peaks ***FK1*** and ***FK2***. Sufficiently pure ***FK1*** and ***FK2*** enabled their identification using NMR and high resolution mass spectrometry as the coniferyl lignin precursors, *erythro-*guaiacylglycerol-8-*O*-4´-(coniferyl alcohol) ether (*e*GGCE) and *threo-*guaiacylglycerol-8-*O*-4´-(coniferyl alcohol) ether (*t*GGCE), respectively, and this was verified by comparison with independently sourced samples of each.

In plants, these molecules are termed neolignans, which are derived from the dimerisation of the lignin precursors called monolignols. Lignin is a heterogeneous high molecular weight polymer and a key structural component of secondary plant cell walls [49, 50]. Three monolignols, namely ρ-coumaryl, coniferyl and synapyl alcohols, are synthesised intracellularly and their lignan dimers are secreted extracellularly. This is consistent with the presence of *e*GGCE and *t*GGCE in extracellular xylem sap fluid. Lignans have been found in a range of plants and shown to have a number of medically important biologically activities such as anti-tumor [51, 52], anti-oxidant [53] and anti-inflammatory activities [54, 55]. Of particular relevance here is the anti-tumor activity of neolignan metabolites, which has been proposed to be due to the anti-angiogenic activity of these molecules [56]. Collectively, these findings suggest that subtle changes in the structure of neolignans can dramatically change their effects on angiogenesis. In fact, we have previously reported that lipo-chitin oligosaccharides behave in a similar manner, the molecules varying dramatically in their pro- or anti-angiogenic activity depending on their structure [36].

*e*GGCE and *t*GGCE induced tube formation using the rat aorta ring assay at μM to nM concentrations. Although this bioassay provided initial valuable information about the overall biological activity, other bioassays portraying the major steps in angiogenesis, including endothelial cell proliferation, migration, tube formation and adhesion to extra-cellular matrix components were utilised to understand the cellular mode of action of *e*GGCE and *t*GGCE. A summary of the results obtained is shown in Table 1. A proliferation assay conducted using HUVEC in the presence of a sub-optimal concentration of bFGF (at 12.5 ng/mL) showed that *e*GGCE and *t*GGCE, in particular, were biologically active in enhancing proliferation over a large concentration range peaking at 5 × 10^−8^ M. In addition, *t*GGCE partially stimulated HUVEC proliferation in the absence of bFGF. This suggests that *t*GGCE may be more active than *e*GGCE. Although *e*GGCE stimulated various aspects of HUVEC and HMEC tube formation in the low μM range it did not significantly affect endothelial cell migration or the binding of endothelial cells to the extracellular matrix components fibronectin or vitronectin. Therefore, it is most likely that *e*GGCE and *t*GGCE work primarily by enhancing vascular tube formation, and may be acting synergistically with bFGF.

**Table 1:** Summary of the effects of the soybean-derived neolignans *e*GGCE and *t*GGCE on various aspects of angiogenesis.

Compound	Angiogenesis	EC Proliferation		EC Tube Formation	EC Adhesion		EC Migration
		-bFGF	+bFGF		Fibronectin	Vitronectin	
*e*GGCE	**+**	**-**	**+**	**+**	**-**	**-**	**-**
*t*GGCE	**++**	**+**	**++**	**++**	NT	NT	NT

EC, Endothelial Cell; NT, not tested. **-**, no effect; **+**, enhancing effect; **++**, highly enhancing effect.

In a parallel study the four stereoisomers of GGCE were synthesised and a paper describing the synthesis of these four compounds was recently published [57]. It was found that all four stereoisomers could enhance endothelial cell tube formation, but *e*GGCE was the least active. Furthermore, the enhanced endothelial cell tube formation induced by the four GGCEs was completely inhibited by PD98059, a flavone that inhibits mitogen-activated protein kinase 1/2 (MEK1/2) and bFGF signalling. These data suggest that the GGCEs enhance angiogenesis via direct activation of the FGFR signalling pathway upstream of MEK1/2.

Thus, this study has successfully identified and characterised two novel pro-angiogenic compounds, *erythro*-guaiacylglycerol-8-*O*-4'-coniferyl alcohol (*e*GGCE), and *threo*-guaiacylglycerol-8-*O*-4'-coniferyl alcohol (*t*GGCE) in soybean xylem sap using a rat aorta bioassay guided fractionation approach. Both *e*GGCE and *t*GGCE could significantly enhance *in vitro* endothelial cell proliferation and tube formation on an artificial ECM, possibly by potentiating the potent mitogen, bFGF, and its downstream signalling. Thus, the coniferyl neolignans *e*GGCE and *t*GGCE represent novel pro-angiogenic molecules with considerable clinical potential.

## Supporting information

S1 Fig UV absorbance of HPLC fractions containing *FK1*, *FK2* and *P6*. ***FK1*** (A) and ***FK2*** (B). UV absorbance spectra were essentially identical with λ_max_ = 265 nm and a shoulder at 298 nm. The UV spectra of ***P6*** (C) was characterised by a λ_max_ at 283 and 343 nm.
(TIF)

S2 Fig NMR Spectra of *FK1* and *FK2*. Overlay of 13C-HSQC spectra for the (A) aliphatic and **(B**) aromatic region, highlighting the similarity of spectra between ***FK1*** (red/pink) and ***FK2*** (blue/green). Along with coupling constants measured from 1D 1H NMR spectra, assignments were easily transferred from ***FK1*** to ***FK2***. Negative peaks (green and pink) indicate CH_2_ resonances.
(TIF)

S3 Fig Positive mode ESI-MS of *FK1* and *FK2*. (A) ***FK1***, *erythro* guaiacylglycerol-8-*O*-4´-(coniferyl alcohol) ether, and (B) ***FK2***, *threo* guaiacylglycerol-8-*O*-4´-(coniferyl alcohol) ether).
(TIF)

S4 Fig Effect of *e*GGCE on HMEC tube formation on Matrigel as measured by circle formation. Microscopic view of isolated endothelial cells cultured for 6 hr on Matrigel (A) in the absence of compound or (B) following addition of 5 × 10^−6^ M *e*GGCE which enhanced circle formation, with examples of completed circles indicated (*). (C) Effect of *e*GGCE, at concentrations from 5 × 10^−6^ M to 5 × 10^−12^ M, on HMEC tube formation, measured as number of completed circles. Control cultures contained the same diluent dilution as the test compounds. Data analysis was performed by Student–Newman–Keuls test after one-way ANOVA comparing each group to control in each treatment. Error bars represent SEM (n = 4).
(TIF)

S5 Fig Time course of HMEC wound healing assay. Wounds were made in HMEC monolayer in each well of a 96-well plate using the IncuCyte wound maker. Cell migration towards the denuded area was recorded every 2 hr and wound recovery was complete within 18–20 hr.
(TIF)

S6 Fig Effect of synthetic *FK1* (*e*GGCE) on HMEC wound healing assay. Synthetic ***FK1***, at concentrations ranging from 5 × 10^−6^ M to 5 × 10^−12^ M, was added to cultures and wound healing was measured as % wound confluence from 0 to 26 hr relative to the initial wound mark. Control cultures contained the same diluent dilution as the test compounds. Data analysis was performed by one-way ANOVA, comparing each group to control. Error bars represent SEM (n = 6). No significant difference in wound healing was observed.
(TIF)

S7 Fig Optimisation of fibronectin and vitronectin concentrations used to coat wells for the Rose Bengal adhesion assay using HMEC. HMEC adhesion to fibronectin (A) and vitronectin (B) was measured as optical density of Rose Bengal staining of adherent cells. Error bars represent SEM (n = 6). The background binding in the absence of fibronectin and vitronectin was < 0.04 optical density unit.
(TIF)

S8 Fig Time course of the effect of synthetic *FK1* (*e*GGCE) on vitronectin-mediated HMEC cell adhesion. Effect of synthetic ***FK1*** on cell adhesion, at concentrations ranging from 5 × 10^−6^ M to 5 × 10^−12^ M, was measured using wells coated with 5 μg/mL of vitronectin and after 2.5–25 min incubation as optical density of Rose Bengal staining adherent cells. Data analysis was performed by one-way and two-way ANOVA, comparing each group to control and other groups. Error bars represent SEM (n = 4). No significant difference in cell adhesion was observed.
(TIF)

S1 Table Comparison of elemental formulas derived from the observed negative ion mode ESI MS/MS spectra with the calculated elemental formulas for *FK1* and *FK2*. (DOCX)

S2 Table Comparison of elemental formulas derived from the observed positive ESI MS/MS spectra with the calculated elemental formulas for *FK1* and *FK2*. (DOCX)

S3 Table Chemical shifts and coupling constants for *FK1* and *FK2*. (DOCX)
