# Supplementary figures and images for: Promotion of mammalian angiogenesis by neolignans derived from soybean extracellular fluids
Source: PLoS One. 2018 May 8;13(5):e0196843. doi: 10.1371/journal.pone.0196843 (PMC5940235; doi:10.1371/journal.pone.0196843)

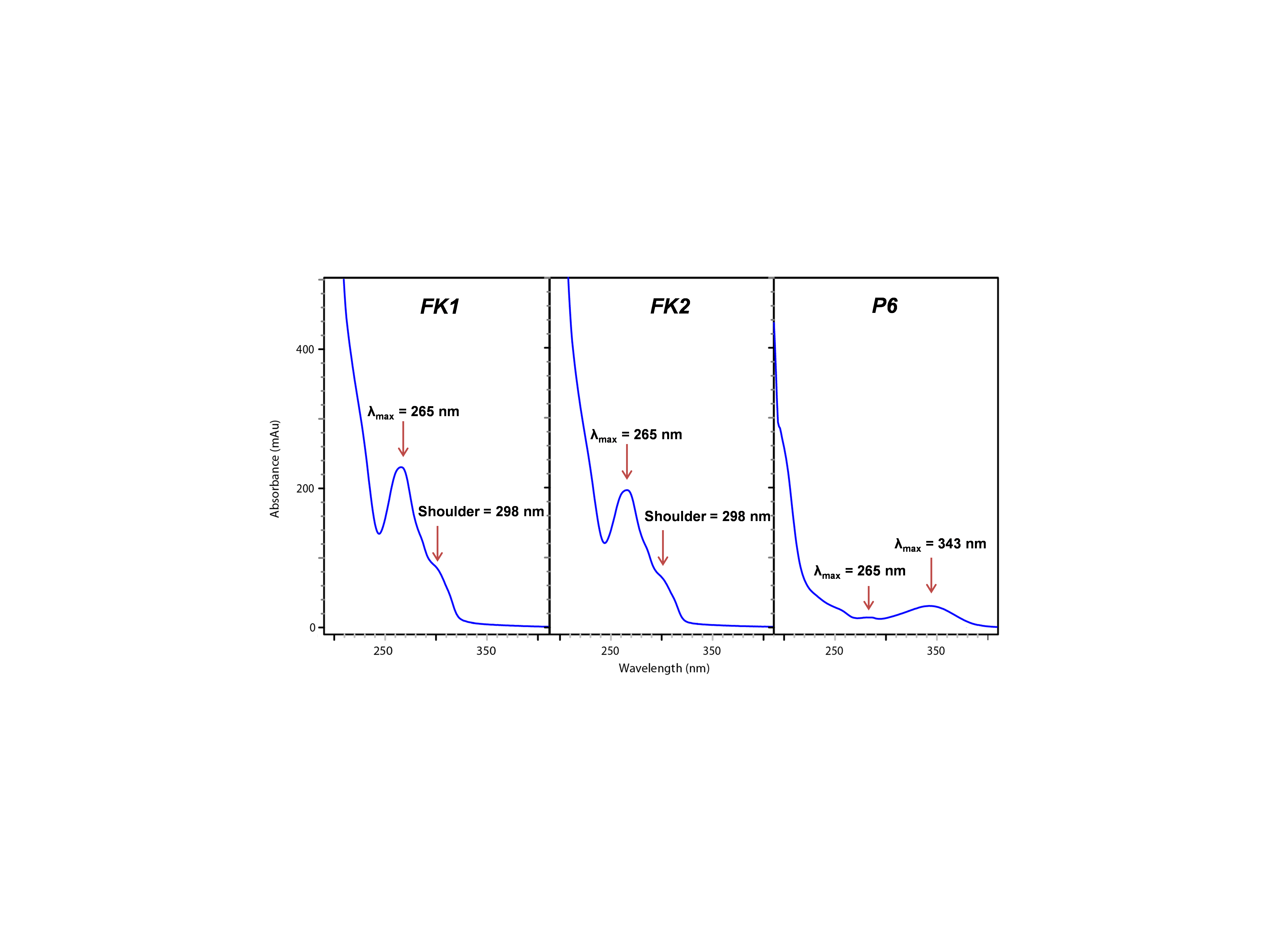

Supplement: S1 Fig — FK1 (A) and FK2 (B). UV absorbance spectra were essentially identical with λmax = 265 nm and a shoulder at 298 nm. The UV spectra of P6 (C) was characterised by a λmax at 283 and 343 nm. (TIF) [file pone.0196843.s001.tif]

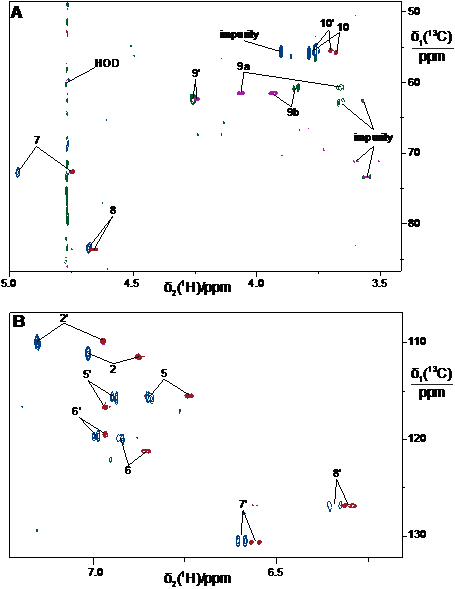

Supplement: S2 Fig — Overlay of 13C-HSQC spectra for the (A) aliphatic and (B) aromatic region, highlighting the similarity of spectra between FK1 (red/pink) and FK2 (blue/green). Along with coupling constants measured from 1D 1H NMR spectra, assignments were easily transferred from FK1 to FK2. Negative peaks (green and pink) indicate CH2 resonances. (TIF) [file pone.0196843.s002.tif]

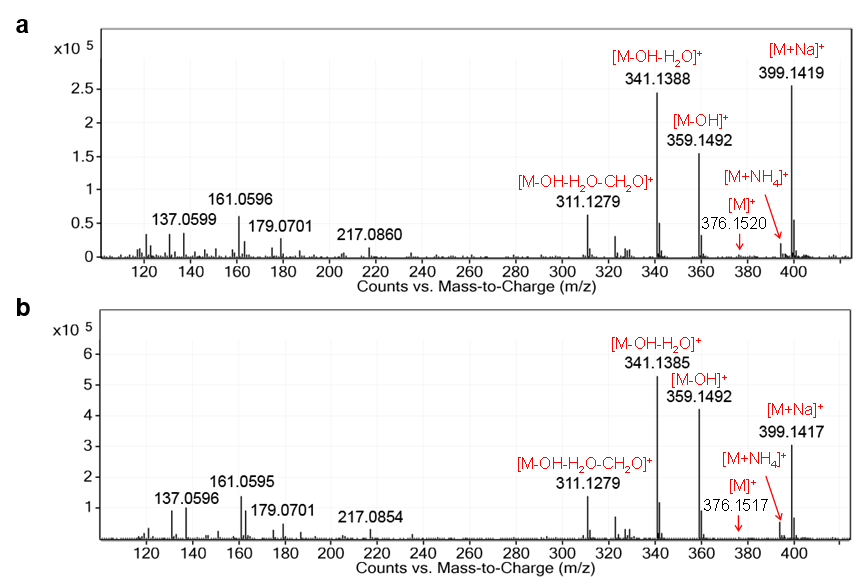

Supplement: S3 Fig — (A) FK1, erythro guaiacylglycerol-8-O-4´-(coniferyl alcohol) ether, and (B) FK2, threo guaiacylglycerol-8-O-4´-(coniferyl alcohol) ether). (TIF) [file pone.0196843.s003.tif]

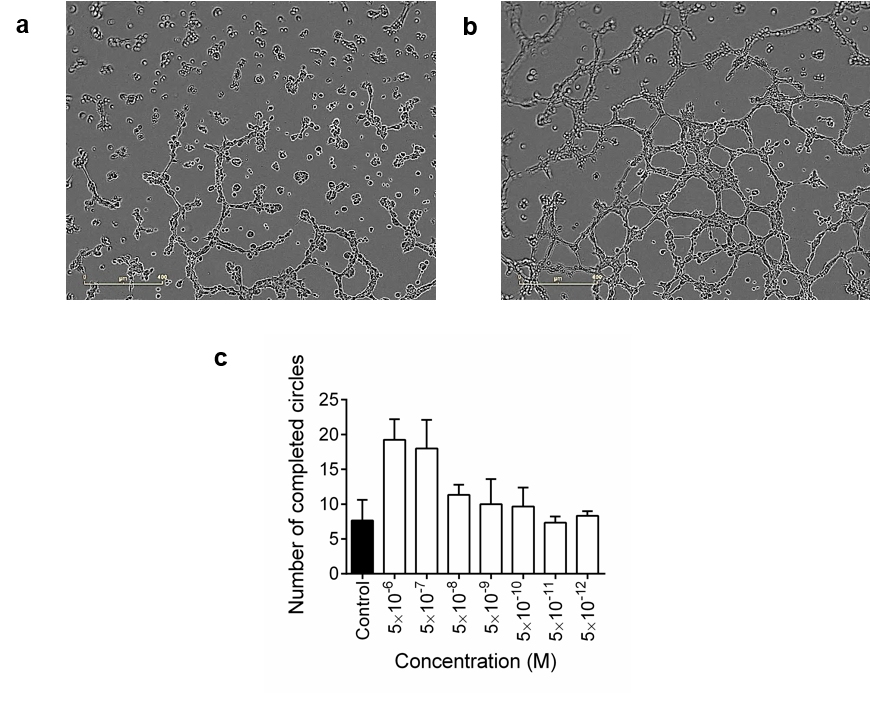

Supplement: S4 Fig — Microscopic view of isolated endothelial cells cultured for 6 hr on Matrigel (A) in the absence of compound or (B) following addition of 5 × 10^−6^ M eGGCE which enhanced circle formation, with examples of completed circles indicated (*). (C) Effect of eGGCE, at concentrations from 5 × 10^−6^ M to 5 × 10^−12^ M, on HMEC tube formation, measured as number of completed circles. Control cultures contained the same diluent dilution as the test compounds. Data analysis was performed by Student–Newman–Keuls test after one-way ANOVA comparing each group to control in each treatment. Error bars represent SEM (n = 4). (TIF) [file pone.0196843.s004.tif]

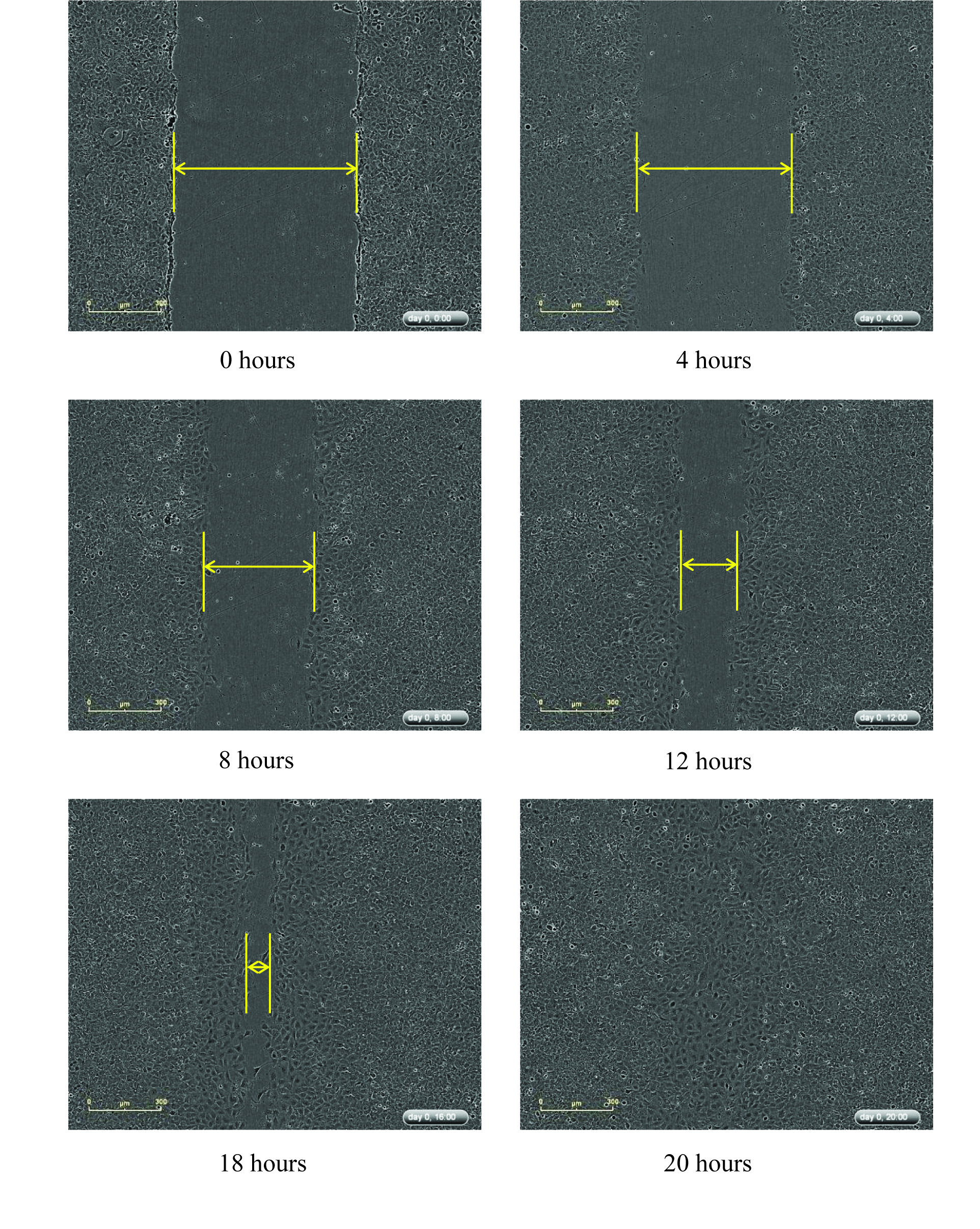

Supplement: S5 Fig — Wounds were made in HMEC monolayer in each well of a 96-well plate using the IncuCyte wound maker. Cell migration towards the denuded area was recorded every 2 hr and wound recovery was complete within 18–20 hr. (TIF) [file pone.0196843.s005.tif]

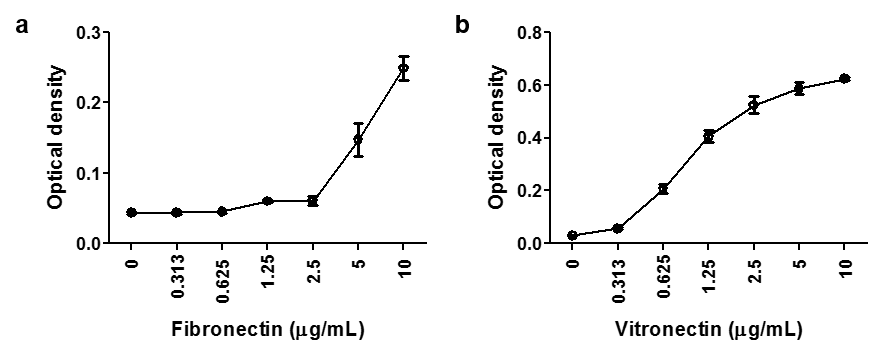

Supplement: S7 Fig — HMEC adhesion to fibronectin (A) and vitronectin (B) was measured as optical density of Rose Bengal staining of adherent cells. Error bars represent SEM (n = 6). The background binding in the absence of fibronectin and vitronectin was < 0.04 optical density unit. (TIF) [file pone.0196843.s007.tif]
