# Supplementary material for: Promotion of mammalian angiogenesis by neolignans derived from soybean extracellular fluids
Source: PLoS One. 2018 May 8;13(5):e0196843. doi: 10.1371/journal.pone.0196843 (PMC5940235; doi:10.1371/journal.pone.0196843)
Supplement: S1 Table — (DOCX) [file pone.0196843.s009.docx]

**S1. Table. Comparison of elemental formulas derived from the observed negative ion mode ESI MS/MS spectra with the calculated elemental formulas for *FK1* and *FK2*.**

| **Product ion** | **Structure** | **Empirical formula** | **Calc *m/z*** | ***m/z*** | | **Δppm** | **Loss** |
| --- | --- | --- | --- | --- | --- | --- | --- |
| [M-H]^-^ | 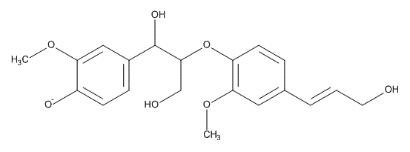 | C_20_H_23_O_7_ | 375.1449 | ***FK1*** | 375.1444 | 1.4 | H |
|  |  |  |  | ***FK2*** | 375.1447 | 0.6 |  |
| [M-H_2_O]^-^ | 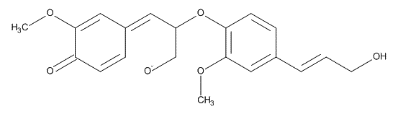 | C_20_H_21_O_6_ | 357.1344 | ***FK1*** | 357.1355 | -3.19 | H_2_O |
|  |  |  |  | ***FK2*** | 357.1351 | -2.07 |  |
| [M-CH_2_O]^-^ | 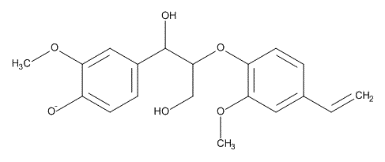 | C_19_H_21_O_6_ | 345.1344 | ***FK1*** | 345.1333 | 3.08 | CH_2_O |
|  |  |  |  | ***FK2*** | 345.1347 | -0.98 |  |
| [M-H_2_O-CH_2_O]^-^ | 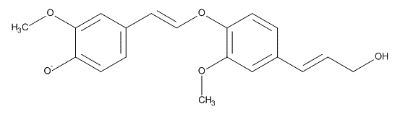 | C_19_H_19_O_5_ | 327.1238 | ***FK1*** | 327.1236 | 0.6 | CH_4_O_2_ |
|  |  |  |  | ***FK2*** | 327.1237 | 0.3 |  |
| [M-H_2_O-CH_2_O-CH_3_]^-^ | 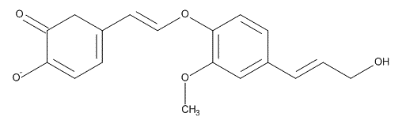 | C_18_H_16_O_5_ | 312.1003 | ***FK1*** | 312.0989 | 4.56 | C_2_H_7_O_2_ |
|  |  |  |  | ***FK2*** | 312.1005 | -0.57 |  |
| A^-^ | 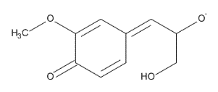 | C_10_H_11_O_4_ | 195.0663 | ***FK1*** | 195.0663 | 0.09 | C_10_H_12_O_3_ |
|  |  |  |  | ***FK2*** | 195.0662 | 0.42 |  |
| [A-CH_2_O]^-^ | 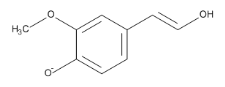 | C_9_H_9_O_3_ | 165.0557 | ***FK1*** | 165.0559 | -1.1 | C_11_H_14_O_4_ |
|  |  |  |  | ***FK2*** | 165.0560 | -1.71 |  |
| B^-^ | 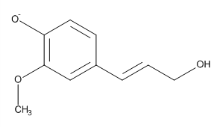 | C_10_H_11_O_3_ | 179.0714 | ***FK1*** | 179.0705 | 4.85 | C_10_H_12_O_4_ |
|  |  |  |  | ***FK2*** | 179.0713 | 0.38 |  |
