# Supplementary material for: Promotion of mammalian angiogenesis by neolignans derived from soybean extracellular fluids
Source: PLoS One. 2018 May 8;13(5):e0196843. doi: 10.1371/journal.pone.0196843 (PMC5940235; doi:10.1371/journal.pone.0196843)
Supplement: S2 Table — (DOCX) [file pone.0196843.s010.docx]

**S2. Table . Comparison of elemental formulas derived from the observed positive ESI MS/MS spectra with the calculated elemental formulas for *FK1* and *FK2*.**

| **Product ion** | **Empirical formula** | **Calc *m/z*** | ***m/z*** | | **Δppm** | **Gain/Loss** |
| --- | --- | --- | --- | --- | --- | --- |
| [M+Na]^+^ | C_20_H_24_O_7_Na | 399.1414 | ***FK1*** | 399.1419 | -1.19 | Na adduct |
|  |  |  | ***FK2*** | 399.1417 | -0.69 |  |
| [M+NH_4_]^+^ | C_20_H_28_O_7_N | 394.1860 | ***FK1*** | 394.1861 | -0.18 | NH_4_ adduct |
|  |  |  | ***FK2*** | 394.1858 | 0.58 |  |
| [M]^+^ | C_20_H_24_O_7_ | 376.1517 | ***FK1*** | 376.1520 | -0.92 | - |
|  |  |  | ***FK2*** | 376.1517 | -0.12 |  |
| [M-OH]^+^ | C_20_H_22_O_6_ | 359.1489 | ***FK1*** | 359.1492 | -0.79 | -OH |
|  |  |  | ***FK2*** | 359.1492 | -0.79 |  |
| [M-OH-H_2_O]^+^ | C_20_H_20_O_5_ | 341.1384 | ***FK1*** | 341.1388 | -1.32 | -H_3_O_2_ |
|  |  |  | ***FK2*** | 341.1385 | -0.44 |  |
| [M-OH-H_2_O-CH_2_O]^+^ | C_19_H_19_O_4_ | 311.1278 | ***FK1*** | 311.1279 | -0.37 | -CH_5_O_3_ |
|  |  |  | ***FK2*** | 311.1279 | -0.37 |  |
