# Supplementary material for: Promotion of mammalian angiogenesis by neolignans derived from soybean extracellular fluids
Source: PLoS One. 2018 May 8;13(5):e0196843. doi: 10.1371/journal.pone.0196843 (PMC5940235; doi:10.1371/journal.pone.0196843)
Supplement: S3 Table — (DOCX) [file pone.0196843.s011.docx]

**S3. Table. Chemical shifts and coupling constants for *FK1* and *FK2***

| ***FK1* Chemical Shifts (*erythro* guaiacylglycerol-8-*O*-4’(coniferyl alcohol) ether)** | |
| --- | --- |
|  | δ(^1^H, D_2_0, 800 MHz) 6.88 (d, ^4^*J*=2.0 Hz, 1H, **H-2**), 6.74 (d, ^3^*J*=8.1 Hz, 1H, **H-5**), 6.86 (dd, ^3^*J*= 8.1 Hz, ^4^*J*=1.9 Hz, 1H, **H-6**), 4.7 (overlapped m, 2H, **H-7**), 4.66 (m, 1H, **H-8**), 4.07 (dd, ^2^*J*= 12.3 Hz, ^3^*J*= 2.8 Hz, 1H, **H-9a**), 3.94 (dd, ^2^*J*= 12.3 Hz, ^3^*J*= 6.7 Hz, 1H, **H-9b**), 3.6824 (s, 3H, **H_3_-10**), 6.98 (bs, 1H, **H-2’**), 6.97 (overlapped s, 1H, **H-5’**), 6.97 (overlapped s, 1H, **H-6’**), 6.56 (dt, ^3^*J*=16.0 Hz, ^4^*J*=1 Hz, 1H, **H-7’**), 6.31 (dt, ^3^*J*=15.9 Hz, ^3^*J*=6.0 Hz, 1H, **H-8’**), 4.25 (dd, ^3^*J*=6.0 Hz, ^4^*J*=1.0 Hz, 2H, **H_2_-9’**), 3.7041 (s, 3H, **H_3_-10’**) |
|  |  |
|  | δ(^13^C, D_2_O, 201 MHz) 130.60 (**C-1**), 111.38 (**C-2**), 147.72 (**C-3**), 147.2 (**C-4**), 115.42 (**C-5**), 121.13 (**C-6**), 72.54 (**C-7**), 83.56 (**C-8**), 61.52 (**C-9**), 55.69 (**C-10**), 131.13 (**C-1’**), 109.78 (**C-2’**), 149.22 (**C-3’**), 146.94 (**C-4’**), 116.56 (**C-5’**), 119.45 (**C-6’**), 130.61 (**C-7’**), 126.76 (**C-8’**), 62.29 (**C-9’**), 55.46 (**C-10’**) |

| ***FK2* Chemical Shifts (*threo* guaiacylglycerol-8-*O*-4’(coniferyl alcohol) ether)** | |
| --- | --- |
|  | δ(^1^H, D_2_0, 800 MHz) 6.94 (d, ^4^*J*=1.9 Hz, 1H, **H-2**), 6.85 (d, ^3^*J*=8.1 Hz, 1H, **H-5**), 6.85 (overlapped dd, ^3^*J*= 8.1 Hz, ^4^*J*=1.7 Hz, 1H, **H-6**), 4.89 (d, ^3^*J*=5.5 Hz, 2H, **H-7**), 4.61 (m, 1H, **H-8**), 3.59 (dd, ^2^*J*= 12.2 Hz, ^3^*J*= 6.0 Hz, 1H, **H-9a**), 3.77 (dd, ^2^*J*= 12.9 Hz, ^3^*J*= 3.7 Hz, 1H, **H-9b**), 3.69 (s, 3H, **H_3_-10** or **10’**), 7.08 (d, ^4^*J*=1.8 Hz, 1H, **H-2’**), 6.87 (d, ^3^*J*=8.64 Hz 1H, **H-5’**), 6.92 (dd, ^3^*J*=8.46 Hz, ^4^*J*=1.8 Hz 1H, **H-6’**), 6.59 (dt, ^3^*J*=15.9 Hz, ^4^*J*=1.0 Hz, 1H, **H-7’**), 6.34 (dt, ^3^*J*=15.8 Hz, ^3^*J*=6.0 Hz, 1H, **H-8’**), 4.18 (dd, ^3^*J*=6.1 Hz, ^4^*J*=1.2 Hz, 2H, **H_2_-9’**), 3.71 (s, 3H, **H_3_-10’** or **10**) |
|  |  |
|  | δ(^13^C, D_2_O, 201 MHz) 110.75 (**C-2**), 115.48 (**C-5**), 119.59 (**C-6**), 72.43 (**C-7**), 83.21 (**C-8**), 60.49 (**C-9**), 55.43 (**C-10**), 109.81 (**C-2’**), 115.42 (**C-5’**), 119.48 (**C-6’**), 130.09 (**C-7’**), 126.70 (**C-8’**), 61.95 (**C-9’**), 55.43 (**C-10’**) |
